# Factors associated with food safety compliance among street food vendors in Can Tho city, Vietnam: implications for intervention activity design and implementation

**DOI:** 10.1186/s12889-022-12497-2

**Published:** 2022-01-14

**Authors:** Ba Huynh-Van, Vy Vuong-Thao, Tuyen Huynh-Thi-Thanh, Sinh Dang-Xuan, Tung Huynh-Van, Loan Tran-To, Nguyen Nguyen-Thi-Thao, Cuc Huynh-Bach, Hung Nguyen-Viet

**Affiliations:** 1 Can Tho University of Medicine and Pharmacy, Can Tho city, Vietnam; 2 Department of Global Development, College of Agriculture and Life Sciences, Cornell University, 14853 Ithaca, New York USA; 3 International Center for Tropical Agriculture, Asia, Vietnam; 4 International Livestock Research Institute, Hanoi, Vietnam; 5 Can Tho City Institute for Socio-economic Development, Can Tho, Vietnam

**Keywords:** Food safety, Street food vendor, Hygiene practice, Street food criteria, Vietnam

## Abstract

**Background:**

Street food plays a valuable role in several Asian countries including Vietnam. Improving the safety of street food is an important responsibility for many local food authorities. This study aims to characterize the business profile of fixed and mobile street food vendors, and to compare their compliance with the food safety criteria.

**Methods:**

A cross-sectional study was conducted using a questionnaire and observational checklist to assess the ten Vietnamese food safety criteria prescribed under Decision No. 3199/2000/QD-BYT for street food vendors in Can Tho city. A total of 400 street food vendors, composed of fixed and mobile vendors, in urban areas of the city were randomly selected for the survey.

**Results:**

The study showed significant differences between the two types of street food vendors in educational level (*p* = 0.017); business profile, including types of foods vended, area in use, number of employees, training in food safety, and business registration paperwork; and the status of compliance with the ten-food hygiene and safety criteria (*p* < 0.01). Poisson regression analysis found that education attainment (IRR = 1.228, *p* = 0.015), food safety training (IRR = 4.855, *p* < 0.01), total business capital (IRR = 1.004, p = 0.031) and total area in use (IRR = 1.007, *p* = 0.001) appeared to be significantly positively associated with food safety and hygiene compliance. In contrast, mobile vending type was negatively associated with the likelihood of adhering to the ten criteria (IRR = 0.547, *p* = 0.005).

**Conclusions:**

These findings emphasize the need for training and education programs to improve food safety knowledge and practice among street food vendors. Basic infrastructure and services, especially clean water, proper sanitation, and waste disposal facilities, should be provided to help street food vendors better practice food safety and hygiene regulations.

**Supplementary Information:**

The online version contains supplementary material available at 10.1186/s12889-022-12497-2.

## Background

Street food is “ready-to-eat” foods and beverages prepared and sold by vendors and hawkers, especially in the streets or public places for immediate consumption [1, 2]. These foods often provide reasonable-price meals, represent a local culture, and generate income with low capital investment for many in developing countries worldwide, such as India, Malaysia, Mexico, Nigeria [3–5]. Vietnamese street food businesses have grown rapidly along with economic growth and urbanization as result of the economic reforms, called “Doi Moi” since 1986. The reforms facilitated increasing rural-urban migration, informal workforce, and non-state-owned food stalls [6, 7]. Street vendors usually join the informal sector because it requires a low capital investment and has negligible taxation [8].

In Vietnam, street food vendors consist of fixed and mobile types, which are categorized based on several available items, such as fixed location, equipment, water, electricity, and type of business registration [9, 10]. Fixed vendors often have fixed stalls, and usually own a business license. In contrast, mobile vendors move from place to place with food on their carts, bicycles, motorcycles, and rarely have a business license. Both street food types have become part of the cultural fabric, serve as important economic opportunities and an affordable food source for Vietnamese people [11, 12].

Despite the above benefits, street foods is considered to be a significant source of foodborne diseases [2, 11]. A recent global report showed that approximate 600 million people, with almost one out of every ten people, get sick and 420,000 deaths result from foodborne illnesses annually [13]. In Vietnam, an average of 180 foodborne disease outbreaks are reported each year, resulting in approximately 6,000 cases of illness and over 40 deaths [14]. However, the actual number could be higher because many cases and small outbreaks are potentially uninvestigated or unreported [13, 14]. The main risk factors that make street food a significant contributor to foodborne illnesses are inadequate infrastructure, improper food handling and poor sanitary conditions at food vending points [2, 5, 15]. Street foods are also often purchased, prepared, and served under several risks of bacteria, virus, pesticide, heavy metals, dust or smoke contamination [16, 17]. In addition, various studies revealed that lack of food safety knowledge, poor attitude towards food safety among vendors, low socio-economic status, and limited food safety regulatory mechanisms were important influences for improper food safety conditions and practices [3, 12, 15, 18].

Given the safety concerns of street food, Vietnam has over time issued several regulations to enhance street food hygiene and safety practices. For instance, Decision No. 3199/2000/QD-BYT standardizes requirements to obtain street food hygiene and safety; Circular 30/2012/TT-BYT specifies food safety conditions for food-catering services and street food businesses and Circular 14/2013/TT-BYT gives guidance on health/medical examination for employees [19–21]. The Decision No. 3199/2000/QD-BYT specifies ten criteria for evaluating street food hygiene and safety practices. Those ten criteria are having adequate clean water; separating raw from cooked food; ensuring hygenic cooking premises; receiving food hygiene and safety training; undergoing health examination; wearing protective clothes; purchasing raw materials from safe and approved suppliers; preparing and displaying food on shelves higher than 60 cm from the ground; storing food in proper containers; and managing waste disposal properly [19].

A 2017 report by the Vietnam Standing Central Inter-Agency Steering Committee on Food Safety revealed that over 20% of street food vendors in Vietnam (approximately 124,000 vendors) did not fully comply with the ten criteria above [22]. Similarly, the rate of non-compliance among street food vendors in Can Tho city is 13.7%, equivalent to 1,048 vendors [22]. There are existing gaps between government regulation requirement and street food vendors’ compliance. Little data on the status and factors associated with street food vendors’ compliance in Vietnam is available. Therefore, this study aimed to assess the current food safety practices of street vendors against the ten criteria and factors associated with this compliance. By considering these gaps and comparing fixed and mobile vendors, the findings provided evidence-based policy recommendations for food safety of street food vendors for Can Tho city, Vietnam, as well as similar contexts in Southeast Asia.

## Methods

### Description of the study area

**Fig. 1:**
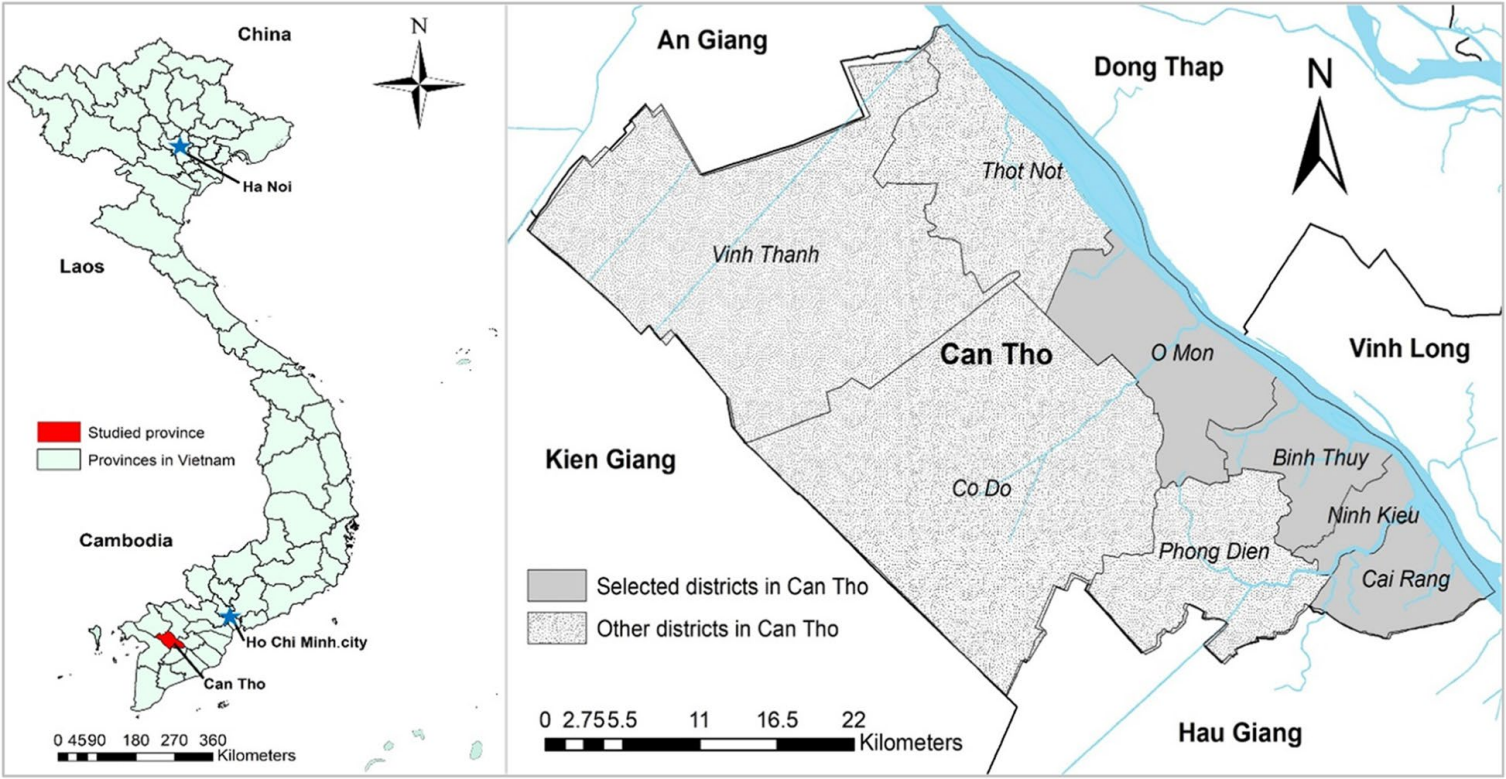
Study districts in Can Tho city in the Mekong Delta region, Vietnam

The study was conducted in Can Tho city, the center of the Mekong Delta, stretching over 55 km along the west bank of Hau river in the South of Vietnam [23]. With an overall land area of 1,439 km^2^, Can Tho city has a population of 1.3 million [24] people residing in four urban districts, one peri-urban district and four rural districts. Because street food vendors operate mainly in the urban districts, we selected four urban districts located in the center of the city as the study sites, namely Binh Thuy, Cai Rang, Ninh Kieu, and O Mon (Fig. 1).

### Study design and sample size

A cross-sectional study was conducted in four urban districts of Can Tho city, Vietnam. The data collection was carried out from October 2017 to January 2018. The sample size was determined by using the single proportion calculation, with 95% confidence level, estimated prevalence of 50% to reach the maximum sample size, and margin of error of 0.05. The required sample size was 384, after accounting for a 5% attrition rate, the final sample size was rounded off as 400 street food vendors.

The number of street food vendors per selected district in the study was proportionally determined according to the total number of street food vendors provided by local governments. from the provided lists, simple random selection was used to obtain the street food vendors list for the survey. Street food vendors participated in the study were then classified into fixed and mobile. Researchers conducted the survey in both day and night time to reach both fixed and mobile street food vendors. (see Supplementary Table S1).

### Questionnaire and data collection

A structured questionnaire and an observational checklist were made in Vietnamese and used to collect information. The questionnaire was developed based on the ten food hygiene and safety criteria for street food vendors in the Decision No. 3199/2000/QĐ-BYT of the Ministry of Health [19]. The questionnaire comprised four main parts. The first part was designed to determine the food vendors’ socio-demographic characteristics including age, gender, educational background, ethnicity, and years of vending experience. The second part assessed food safety knowledge and attitude in 13 sub-sections with a total of 39 yes-no and multiple-choice questions. The third part focused on the food safety and hygiene practice of vendors with 27 questions under seven sub-sections. The third part also included an observation checklist of items which assessed business type, duration of vending, registration status (i.e. license), food safety and hygiene practices, and vendor’s facilities, etc. The fourth part covered information about number of employees, food safety training and any comments of vendors. The questionnaire and checklist can be found in Supplementary Table S2 and S3.

The questionnaire and checklist were pretested among ten street food vendors in a similar study context (urban) for validationbefore a final version was administered to street food vendors. The interview and observation were carried out in-person by trained enumerators. Street food vendors who stopped operating during the data collection period or those who refused to participate were replaced by the nearest and same-categorized vendors. All data collection was carried out in accordance with relevant guidelines and regulations.

### Data analysis

Data were entered in EpiData software version 3.1 (The EpiData Association, Odense, Denmark) and analyzed by SPSS software version 19.0 (SPSS Inc., Chicago, IL, USA). The numerical data were analyzed for descriptive statistics. For inferential analysis to compare the difference between fixed and mobile vendors, T-test and ANOVA were used to test for differences of continuous variables, while the Chi-square test was used to compare proportions among groups. Poisson regression analysis was used to determine the factors associated with vendors’ compliance with the ten food safety criteria. Poisson regression was chosen because the dependent variable are discrete that is, they take on a finite number of mutually exclusive non-negative integer values. Specifically, it’s the compliance score ranging from zero to ten, reflecting the number of criteria (out of ten) achieved by the vendors. A bivariate analysis was first performed to identify potential predictor variables. Variables with a p≤0.25 according to the bivariate analysis and variables that were considered to be important confounders in previous literature were included in the multivariate analysis. A stepwise selection procedure was used to simplify the models. Two multivariate models were applied to determine the factors influencing the compliance with the ten food safety and hygiene criteria among the respondents. Model 1 included age, education level, total business capital, total area in use, mobile vending type and duration of business operation as predictors. Model 2 included all predictors in Model 1 plus food safety training participation. Type of vendors was treated as a predictor variable to avoid the sparseness of data (stratification would reduce the sample size, which might have negatively impacted the performance of the models) [25] and to avoid computational complexities given that we had to include, simultaneously, other covariates and confounders in the analysis. All analyses were carried out with a significance level of 5% and all tests were two-sided.

## Results

### Socio-demographic characteristics of street food vendors

Out of 400 respondents, the number of fixed and mobile vendors was 254 (63.5%) and 146 (36.5%), respectively. In both vendor types, females were predominant and constituted 73.8% of all respondents. About one-fourth of the respondents were between 17 and 35 years old, and over half (56%) of vendors were in middle-age range, 36-55 years old. Almost 54% of fixed vendors had attained senior secondary school or higher education, while 36.3% of mobile vendors had achieved that education level. There was significantly higher education level in the fixed vendors group compared to the mobile vendor group (p=0.017). More than one-third (34.9-39.0%) of both groups have sold street food for more than 5 years, whereas 86/400 (21.5%) had less than one year experience (Table 1).

**Table 1:** Socio-demographic characteristics of the respondents

Characteristics	Overall (*n*=400 (%))	Fixed (*n*=254 (%))	Mobile (*n*=146 (%))	*p*-value
**Gender**				0.528
Female	295 (73.8)	190 (74.8)	105 (71.9)	
Male	105 (26.2)	64 (25.2)	41 (28.1)	
**Age (years)**				0.446
17 - 25	25 (6.3)	18 (7.1)	7 (4.8)	
26 - 35	93 (23.3)	62 (24.4)	31 (21.2)	
36 - 45	119 (29.7)	68 (26.8)	51 (34.9)	
46 – 55	107 (26.7)	68 (26.8)	39 (26.7)	
> 55	56 (14.0)	38 (14.9)	18 (12.4)	
**Education** ^a^				0.017
Illiterate	31 (7.7)	21 (8.3)	10 (6.9)	
Primary school	51 (12.7)	26 (10.2)	25 (17.1)	
Junior secondary school	129 (32.3)	71 (28.0)	58 (39.7)	
Senior secondary school	124 (31)	88 (34.6)	36 (24.7)	
College	25 (6.3)	19 (7.5)	6 (4.1)	
University	40 (10)	29 (11.4)	11 (7.5)	
**Work experience**				0.625
Under 1 year	86 (21.5)	56 (22.0)	30 (20.6)	
1 to < 3 years	104 (26.0)	65 (25.6)	39 (26.7)	
3 to < 5 years	60 (15.0)	34 (13.4)	26 (17.8)	
5 years and above	150 (37.5)	99 (39.0)	51 (34.9)	

^a^Education level referred to primary school (grade 1-5, 6-10 years old [yo]), senior secondary school (grade 6-9, 11-14 yo); senior secondary school (grade 10-12, 15-17 yo); college/university (18 yo and above)

### Business profile of street food vendors

About 64% of the vendors prepared food and sold on site, and the rest (36%) sold ready-to-eat food. Soups, such as noodle soup and ‘*pho*’ (beef noodle soup) were the most popular food types sold by fixed vendors (39.4%), while only 16.4% of mobile vendors sold this type of food. Easy-to-takeaway food such as rice paper salad, traditional rice cakes of all types, fried fish/beef/chicken/tofu, spring rolls, and dumpling, etc. were the most common food served by mobile vendors. Beverages (coffee, soft drinks) were also common among both fixed and mobile vendors (17-21%, Table 2).

The average number of years operating business was six, ranging from 0.5 up to 50 years, and there was no difference in number of years of business operation between fixed and mobile vendors. The average area in use was significantly different between fixed vendors and mobile vendors, 44.2 m^2^ and 11.3 m^2^, respectively. Fixed vendors, on average, had more employees than mobile ones, four and two employees per business, respectively (Table 2).

The proportion of obtaining required documents (certificates) was significantly different between fixed and mobile vendors (*p* <0.001). While 33.1% of the fixed vendors owned a business registration license, only 8.2% of mobile vendors got a license. The similar patterns were observed for food safety requirement, training or health check certificates (Table 2).

**Table 2:** Characteristics of fixed and mobile vendors

Characteristics	Overall (*n*=400)	Fixed (*n*=254)	Mobile (*n*=146)	*p*-value
**Cooking and serving points** (n, %)				0.862
Prepare and cook food to sell onsite	257 (64.3)	164 (64.6)	93 (63.7)	
Sell ready-to-eat food directly	143 (35.8)	90 (35.4)	53 (36.3)	
**Types of food vended** (n, %)				
Soup (noodle, rice soup etc.)	124 (31.0)	93 (36.6)	31 (21.2)	0.004
Sticky rice^a^	60 (15.0)	48 (18.9)	12 (8.2)	0.004
Fired food, rice cake, dumpling, spring roll^b^	72 (18.0)	34 (13.4)	38 (26.0)	0.002
Sausage, fermented pork, kebub/bread	33 (8.3)	20 (7.9)	13 (8.9)	0.915
Coffee, soft drink, ice cream	77 (19.2)	40 (15.7)	37 (25.3)	0.072
Dessert, fruits	18 (4.5)	7 (2.8)	11 (7.6)	0.534
Ice	5 (1.3)	3 (1.2)	2 (1.4)	0.625
Beer, wine…	11 (2.7)	9 (3.5)	2 (1.4)	0.156
**Year of operation and employees**				
Years of operation (Mean ± SD)	6.4±0.3	6.4±0.4	6.5±0.6	0.702
Area in use (m^2^, Mean ± SD)	32±2.3	44.2±3.2	11.3±2.0	0.000
Number of employees (Mean [min-max])	3 (1-12)	4 (1-12)	2 (1-6)	0.000
Trained in food hygiene and safety (person, mean [min-max])	2 (1-9)	2 (1-9)	2 (1-4)	0.003
**Having related documents (n, %)**				
Business registration license	96 (24.0)	84 (33.1)	12 (8.2)	0.000
Food safety requirements certificate	104 (26.0)	89 (35.0)	15 (10.3)	0.000
Food safety training certificate	127 (31.8)	99 (39.0)	28 (19.2)	0.000
Health check certificate	142 (35.5)	107 (42.1)	35 (24.0)	0.000

^a^with nuts or corn, or toping with green bean powder, fried onion, fried egg, caramel pork, etc.; ^b^such as: mixed rice paper, traditional rice cakes, fried pork, (fish, beef, chicken, tofu), spring roll, sausage, dumpling

### Street food vendors facilities and food safety practices

Food hygiene and safety practices among study respondents highlighted significant disparities between fixed and mobile vendors, where the fixed vendors performed better than the mobile vendors in almost all categories (Table 3). Results showed that 93.7% of fixed vendors and 85.6% of mobile vendors processed or stored food on tables or cabinets at least 60 cm above the ground and 92.9% of fixed vendors had sufficient waste bins with properly closed lids, this percentage among mobile vendors was 71.2%. Up to 85.8% of fixed vendors regularly used plastic bags instead of newspapers, used or recycled paper, to package food while 76.7% of mobile vendors sometimes followed this practice. There was a significant difference in access to clean water at food vending sites between fixed and mobile vendors with 99.2% of fixed vendors and 67.4% of mobile vendors supplying their vending sites with enough clean water, respectively.

Regarding food safety practices, only 15/254 (5.9%) fixed vendors and 2/146 (1.4%) mobile vendors stored food samples at 4-8^o^C for 24 h as requirement interm of rfood safety investigation. Only 72/254 (28.4%) fixed vendors and 26/146 (17.8%) mobile vendors were observed to wear face masks. Over 68% of vendors used separate cutting boards for raw and cooked foods, however, only two fifth (41%) of vendors had handwashing facilities at their places (Table 3).

**Table 3:** Street food vendors facilities and food safety practices

Characteristics	Overall (*n*=400 [%])	Fixed (*n*=254 [%])	Mobile (*n*=146 [%])	*p*-value
Tables/cabinets were at least 60 cm height from the ground	363 (90.8)	238 (93.7)	125 (85.6)	0.035
Have enough clean water at vending site	296 (74.0)	234 (99.2)	62 (67.4)	0.000
Have handwashing facility (e.g. sink, bucket, or washbasin with soap)	164 (41.0)	121 (47.6)	43 (29.5)	0.000
Have origin information and invoice of raw food materials	139 (34.8)	107 (42.1)	32 (21.9)	0.000
Have waste bins with closed lids	340 (85.0)	236 (92.9)	104 (71.2)	0.000
Food handlers wear gloves during preparing and cooking food	205 (51.3)	159 (62.6)	46 (31.5)	0.000
Food handlers wear mask during selling	98 (24.5)	72 (28.4)	26 (17.8)	0.012
Food handlers wash hands during selling	271 (67.8)	187 (73.6)	84 (57.5)	0.000
Use separate chopstick, tongs to handle raw and cooked meat	231 (57.8)	162 (63. 8)	69 (47.3)	0.001
Use separate cutting boards for raw meat and cooked foods	160 (40.0)	118 (46.5)	42 (28.8)	0.000
Use separate containers to store raw and cooked foods	172 (43.0)	132 (52.0)	40 (27.4)	0.000
Use clean bags/boxes to package foods	330 (82.5)	218 (85.8)	112 (76.7)	0.016
Reuse cooking oil during selling	49 (22.4)	28 (18.7)	21 (30.4)	0.040
Apply storing food samples for inspection^a^	17 (4.3)	15 (5.9)	2 (1.4)	0.022

^a^This requirement is not included in the ten-food safety and hygiene criteria. However, the Decision No. 1246/QD-BYT, 2017 requires food sellers to store food samples at 4-8^o^C for at least for 24 h for investigation purpose (if any), which is required for food service establishments

### Status of compliance with the ten-food safety criteria

**Fig. 2:**
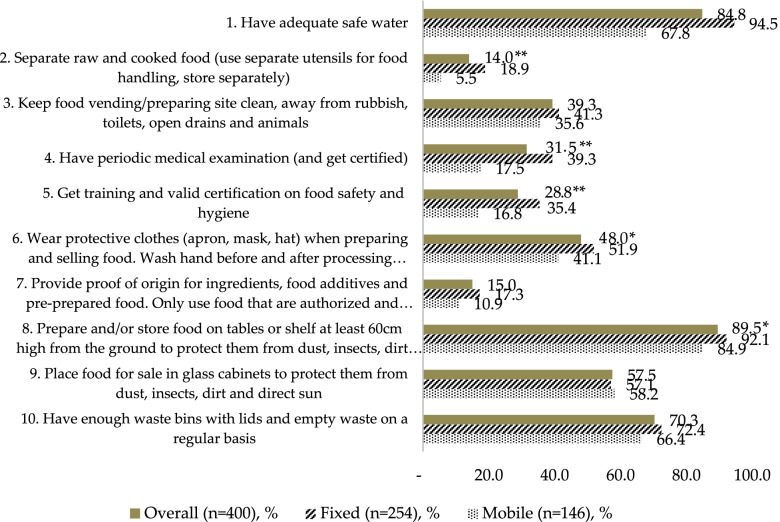
Compliance rate by each of the ten food safety criteria between fixed and mobile vendors

Among fixed food vendors, the two criteria that were fulfilled by over 90% of vendors were having adequate clean water (criterion 1) and protecting food from dust, insects, and direct sun by placing it on tables or shelves at least 60 cm above the ground (92.1%, criterion 8). The proportion of mobile food vendors achieving these two criteria was 67.8% and 84.9%, respectively. The two criteria that have the lowest compliance rate, below 20% by both vendor types, were separating raw and cooked food (criterion 2) and sourcing food from safe and approved suppliers (criterion 7). Fixed vendors had a significantly higher compliance rate, for instance criteria No. 1, 2, 4, 5, 6 and 8 compared to those of mobile vendors. However, for both vendor types, only four out of ten criteria were higher than 50% (Fig. 2).

### Factors associated with achieving food safety criteria of street food vendors

From the bivariate analysis, a significant association was found between the compliance status with age, education level, total business capital, total area in use for business, mobile vending type and training in food safety (*p* < 0.01), whereas gender and duration of business operation were not significantly associated with the compliance status of respondents on food safety and hygiene practices (*p* > 0.05, Table 4).

Multivariate Poisson regression showed that the compliance with the ten food safety and hygiene criteria was associated with education level, total business capital, total area in use for business and mobile vending type (Model 1, Table 4). These factors, together with food safety training, continued to be significantly associated with compliance with the ten food safety and hygiene criteria (Model 2, Table 4). Both multivariate models were statistically significant with *p* < 0.01 and R^2^ equal to 20.9% and 34.4%, respectively.

Specifically, education level, total business capital, total area in use and food safety training were positively associated with the ability to comply with the ten criteria, while mobile vending type was negatively associated with the compliance. Vendors with higher education level are more likely to achieve the ten criteria for food safety (*p* = 0.015). The compliance score of vendors who received food safety training was almost five times higher than that of vendors who did not receive food safety training (IRR = 4.855, *p* < 0.0001). Mobile street vendors regularly had 0.547 times compliance score compared to fixed vendors (IRR = 0.547, *p* = 0.005). Total business capital and total area in use, though they achieved statistically significant *p*-value, had a smaller effect on the compliance status than education level, food safety training and mobile vending type.

**Table 4:** Potential factors affecting the compliance of street food vendors to ten food safety and hygiene criteria

Items	Beta	IRR	SD	t-value	*p*-value
**Bivariate model**					
Gender					0.976
Age					0.002
Education level (category variable)					0.000
Total business capital					0.000
Total area in use for business					0.000
Mobile street food vendor					0.000
Duration of business operation					0.205
Receiving food safety training					0.000
**Multivariate model 1** ^a^					
Constant	3.910	49.899	0.570	6.866	0.000
Age	-0.121	0.886	0.109	-1.111	0.268
Education level	0.284	1.328	0.091	3.108	0.002
Total business capital	0.006	1.006	0.002	2.992	0.003
Total area in use for business	0.010	1.010	0.003	3.416	0.001
Mobile street food vendor	-0.628	0.534	0.234	-2.686	0.008
Duration of business operation	0.007	1.007	0.018	0.388	0.698
***R***^***2***^		20,9%			
***p*** **-value**		0.000			
**Multivariate model 2** ^b^					
Constant	3.237	25.457	0.527	6.144	0.000
Age	-0.093	0.911	0.099	-0.940	0.348
Education level	0.205	1.228	0.084	2.444	0.015
Total business capital	0.004	1.004	0.002	2.171	0.031
Total area in use for business	0.007	1.007	0.003	2.604	0.010
Mobile street food vendor	-0.604	0.547	0.213	-2.830	0.005
Duration of business operation	0.004	1.004	0.016	0.246	0.806
Receiving food safety training	1.580	4.855	0.205	7.700	0.000
***R***^***2***^		34.4%			
*p* **-value**		0.000			

^a^Adjusted for age, education level, total business capital, total area in use, type of vendor and year of business operation; ^b^Adjusted for all variables in Model 1 plus Awareness of the need for food safety training; *IRR* Incidence Rate Ratio; *SD* Standard Deviation

## Discussion

The current study highlighted significant differences in socio-demographics, business profile and participation in food safety training, and thus the status of fulfilling the ten food safety and hygiene criteria between fixed and mobile vendors in Can Tho city, Vietnam. The study also identified the factors that were associated with complying with these ten criteria. Education level, food safety training and mobile vending type had greater impacts on the compliance status than the total business capital and total area in use. The results of this study suggested potential approaches to promote food safety and hygiene practices among street food vendors in Vietnam.

### Difference in socio-demographics and business profile between fixed and mobile vendors

Senior secondary school was the most common education level among fixed vendors while junior secondary school-level education prevailed among mobile vendors. The proportion of fixed vendors with a higher educational level was higher than the educational status of street food vendors in the previous studies conducted in Vietnam [12, 26, 27], and studies in Ghana [16], Uganda [28], and India [17]. The results from these studies showed that street food vendors had a relatively low level of education, with primary or junior secondary school as the most prevalent level, which was similar to that of mobile vendors in this study.

Having stable facilities gave fixed vendors preferable conditions in terms of diversity of food vended, larger area in use and more employees. Another trait distinguishing fixed and mobile vendors was formal training in food hygiene and safety. Specifically, on average, there were more fixed business employees receiving training than mobile business employees. Street food vendors should participate in food safety and hygiene training at least once a year to update and improve knowledge and practice in this field [9]. Lack of training in food safety was found to be associated with poor hygiene practices [9, 29].

The largest difference between the two types of vendors was the food vending-related documents. More fixed vendors obtained those documents, such as business registration licenses, food safety requirements certificates, food safety training certificates, or health check certificates. However, the general proportion of vendors, regardless of types, holding the above-mentioned documents was considerably low, less than 40% of the total study respondents. Food-related business is often a “conditional” business requiring owners or persons involved to fulfill certain facility conditions and acquire required certificates (e.g. separation of equipment for raw and ready-to-eat food, training on hygiene practices, and health check) to operate their street food vending (Decree 155/2018/ND-CP) [30]. So far, the process of guiding, harmonizing, and monitoring of food safety standards by local authorities, as well as ensuring food vendors comply with the various food safety requirements, circulars and decrees need to be improved.

### Difference in food safety practices and the compliance status with the ten food safety and hygiene criteria between fixed and mobile vendors

Most fixed vendors had a higher rate of complying with each of the ten criteria than mobile vendors. Fixed vendors achieved the highest compliance rate in having sufficient clean water and cool storage containers (94.5%). This result is slightly lower than that found by Toan Luu [27] in Hanoi, Vietnam, where 100% of food vendors complied with having clean water. Meanwhile, only 67.8% of mobile food vendors in this study satisfied this criterion. The Codex Alimentarius indicated that the provision of sufficient clean water at food vending points was crucial for vendors to wash hands and used bowls and utensils regularly [15]. However, street vendors in various parts of the world were reported to wash their utensils in water that has been used repeatedly [31], probably due to the cost or unavailability of clean water [28]. This might explain the lower compliance rate of mobile vendors who could not equip themselves with adequate clean water due to inconveniences in accessing water sources and their mobility.

As a result of having sufficient water at the vending points, the rate of proper handwashing by fixed vendors was considerably higher than that of mobile vendors (73.62% versus 57.53%). Hands are the significant vehicle for the transmission of disease-causing organisms such as *Salmonella*, *Campylobacter* and *Escherichia coli*, from feces, nose, skin or other surfaces to food [28, 29]. Studies in Nigeria and Kenya found that most of the food vendors did not have access to toilet facilities when vending on the street [29, 32]. These vendors had to make use of secluded areas in place of a public toilet and some of them rarely washed their hands after using the toilet [32]. Without sufficient clean water and washing/sanitation facilities, proper hygiene is hardly put into practice by street food vendors [12].

Another mechanism for avoiding pathogen transmission is separating raw food from cooked food. Only 20% of fixed vendors and 6% of mobile vendors met this food safety criterion. These proportions were lower than those in Haiti, where 100% of the vendors stored raw and cooked food separately [33], Malaysia, where around 95% did the same [34], and Vietnam, 47.5% [12]. This study’s results were comparable to the results of two studies in Ghana [16, 35] and Nigeria [9] where 25–27% of food vendors separated raw food from cooked food. This poor practice has led to food contamination with microorganisms and could cause foodborne illness from consuming cooked food directly [16]. Therefore, the WHO International Food Safety Authorities Network (INFOSAN) states the separation of raw and cooked food as one of the “Five keys to safer food” for food sector [2, 36].

On the other hand, the origin and specifications of raw materials are among the most pressing food safety issues in Vietnam, especially in relation to the street food sector. In this study, only 17.3% of fixed vendors and 10.9% of mobile vendors were able to provide proof of raw materials’ origin and specifications (criterion 7). This criterion helps to make sure the materials are safe, such as by certifying that foods have no prohibited additives or restricted substances, are not expired, and meat products have been inspected. However, vendors usually keep the input costs low by using low-quality, cheap ingredients, dubious sources [37], or even using banned or excessive chemicals and additives to hide the poor-quality materials [38]. For example, a study in Nigeria found that more than 90% of vendors prioritized quantity and price over freshness and cleanliness when purchasing raw materials [32].

One of other criteria is medical examination (health check) that helps to detect and treat carriers of harmful pathogens among food handlers to reduce the risk of food contamination, and consequently to protect consumers [39]. In Vietnam, street food handlers and processors are expected to carry out a physical examination, and stool and urine tests to obtain and renew their health certificate annually [20, 33, 40]. Only 39.3% of fixed vendors and 17.5% of mobile vendors in this study had a health certificate. However, evidence indicates that medical examinations can be a financial burden for vendors and, overall, an ineffective tool because they are given after a one-time examination and cannot prevent infection after the examination is performed [26, 41].

### Factors associated with the ten food safety criteria compliance among street food vendors

This study found that education, training in food hygiene and safety, type of food vendor, total business capital and total area in use were significantly associated factors with food safety compliance. Level of education and training have been reported to play an important role in increasing the awareness of food safety of food handlers, thus enhancing their attitudes and practices towards food safety and hygiene [1, 3, 13]. The findings from this study revealed that vendors who completed senior secondary school and above, or those participating in food safety training, satisfy more criteria i.e., they had better food safety practices. Training was confirmed to be significantly associated with improving food safety practices in studies in Nigeria [9, 32], South Africa [42], and Malaysia [34]. However, a few other studies in India [17] and Ghana [35] showed that education level had no significant impact on either food safety knowledge or practice.

The ability to satisfy minimum requirements for maintaining hygienic practices is also positively related to the business capital and the number of employees of the street food entrepreneurs as found in this study. Fixed food vendors are more likely to have proper facilities and equipment for business operations, including storage equipment, safe cooking tools, hand and utensils’ washing facilities, and waste disposal systems [12, 32]. A recent initiative of the local government in Can Tho is gathering street food vendors into ‘street food centers’ to provide them with basic facilities such as kiosks, piped water, electricity, and toilets. Then vendors can hire and use these facilities at a fee. A previuos study in urban Hanoi, Vietnam emphasized the food safety concerns and “supermarketization” policies that are seeking to reduce the amount of informal food vendors and increase formal retail outlets [43], however, the role of street food vendors are still important to vendors’ livelihood and contribute to food accessibility of consumers during the economic growth transition and maintain food cuisine culture.

This study also showed that the more employees a street food business had, the higher chance that the business would follow food safety practices. This might be explained by the ability of such establishments to share workload among employees. If one person was not overloaded with preparing and selling food, and handling money from customers at the same time, they might have the capacity to better follow food safety practices. A study in Ho Chi Minh City, Vietnam, observed that 70% of vendors used their bare hands to exchange money while handling and serving food [12], and the similar poor practice was also reported in Uganda [28] and Ghana [16]. Money has been shown to harbor foodborne pathogens such as *Salmonella*, *Staphylococcus aureus* [28] since it passes through the hands of many people [16].

Street vendor mobility could be a predictor of food safety compliance. This study found that mobile vendors had a lower chance of satisfying the ten food hygiene and safety criteria than fixed vendors. In addition, fixed vendors were more likely to hold a business license or permit. This legal status might give access to social and legal protection and necessary services and facilities, which in turn would help protect vendors from certain risks, including confiscation of merchandise or being removed completely from the streets [38] and help them to better adopt food safety practices. This study was quantitative in nature, we could not gather the qualitative data (e.g. through focus group discussions or in-depth interviews) to get vendors perspectives on food safety and how and why they do or do not adhere to the established regulatory criteria. This is a potential future research area, which would further refine this study’s findings and strengthen the policy, program changes suggested.

### Study strengths and limitations

To our knowledge, this is the first study to evaluate and compare fixed and mobile street food vendor business characteristics and their compliance with the ten food safety criteria in Vietnam. The study’s findings can help to customize supporting policies for different types of street food vendors to improve food safety in the country. Further, our measurement tools were piloted resulting in their increased reliability. However, this study had some limitations. First, the cross-sectional nature of study design prevented us from making any causal conclusions between variables. Second, some explanatory variables relied on participants’ self-reported data, which were prone to recall bias and social desirability bias. To minimize this bias, study participants were allowed adequate time to try to recall previous information as best as they could. We also asked participants to provide us with records of some food safety practices such as certification of medical examination and certification of food safety training. Finally, the observation of food safety and hygiene practices can lead to the Hawthorne effect, the awareness of being studied and thus potential influence on behavior of study participants [44].

## Conclusions

This study assessed the differences in socio-economic status, business profile, food safety training and status of compliance with the ten food hygiene and safety criteria between fixed and mobile vendors, where the former performed better. The results also indicated that education attainment, training in food safety, business capital, number of employees and type of vendors had a significant relationship with the compliance status. These findings suggested a need to develop policies and programs to support street food businesses, which are an important food and income source for a growing urban population in Vietnam. Street food vending should be formally recognized as part of the national food system and covered by urban development programs if possible [45]. In addition, regulations and support should be appropriate to better enable street food vendors adhere to food safety standards, which would, in turn, save the human and financial resources of local authorities. Current regulations on food safety for street vendors can be made more effective in practice by utilizing incentives such as subsidies for equipment procurement, microcredit programs, food safety compliance awards, raising awareness of nutrition and food safety via training, education programs and public media, and imposing sanctions on violating minimum food safety standards. It is also crucial to engage all stakeholders, vendors, customers, local authorities, and non-profit organizations in these interventions to ensure a fair ground where all voices are heard, which, in turn, will encourage stakeholders to become more committed to complying with food safety standards.

## Supplementary Information

**Additional file 1: Figure S1.**

## Data Availability

The datasets used and analyzed during the current study are available from the corresponding author on reasonable request.
