# Supplementary material for: Factors associated with food safety compliance among street food vendors in Can Tho city, Vietnam: implications for intervention activity design and implementation
Source: BMC Public Health. 2022 Jan 14;22:94. doi: 10.1186/s12889-022-12497-2 (PMC8759169; doi:10.1186/s12889-022-12497-2)
Supplement: Supplementary file 1 — Additional file 1: Figure S1. [file 12889_2022_12497_MOESM1_ESM.docx]

**Factors Associated with Food Safety Compliance among Street Food Vendors in Can Tho City, Vietnam: implications for intervention activity design and implementation**

Ba Huynh-Van^1, *^, Vy Vuong-Thao^2,^, Tuyen Huynh-Thi-Thanh^3^, Sinh Dang-Xuan^4^, Tung Huynh-Van^5^, Loan Tran-To^5^, Nguyen Nguyen-Thi-Thao^5^, Cuc Huynh-Bach^1^, Hung Nguyen-Viet^4^

*^1^Can Tho University of Medicine and Pharmacy, Vietnam*

*^2^ Department of Global Development, College of Agriculture and Life Sciences, Cornell University, Ithaca, New York 14853, USA*

*^3^ International Center for Tropical Agriculture, Asia, Vietnam*

*^4^ International Livestock Research Institute, Hanoi, Vietnam*

*^5^ Can Tho City Institute for Socio – economic Development, Vietnam*

*Corresponding author

Ba Van Huynh

Address: 14/14-Ly Tu Trong Street-Ninh kieu District, Can Tho City, Vietnam

Organization: Can Tho University of Medical and Pharmacy, Can Tho, Vietnam

Phone: +84 908 831414

Email: [bs.ba_fob@yahoo.com.vn](mailto:bs.ba_fob@yahoo.com.vn); [Drbafob64@gmail.com](mailto:Drbafob64@gmail.com)

**Supplementary Materials**

***Table S1: Sampling by district***

| **No.** | **District** |  | **Total street food vendors** | **Selected** | **Number/district** |
| --- | --- | --- | --- | --- | --- |
| 1 | Ninh Kieu | Fixed vendor | 944 | 156 | 250 |
|  |  | Mobile vendor | 1098 | 94 |  |
| 2 | Cai Rang | Fixed vendor | 91 | 36 | 50 |
|  |  | Mobile vendor | 349 | 14 |  |
| 3 | OMon | Fixed vendor | 234 | 24 | 50 |
|  |  | Mobile vendor | 165 | 26 |  |
| 4 | Binh Thuy | Fixed vendor | 70 | 38 | 50 |
|  |  | Mobile vendor | 311 | 12 |  |
|  | Total |  | 3262 | 400 |  |

***Table S2: Interview questionnaire***

| ID: |  |  |  |  |  |
| --- | --- | --- | --- | --- | --- |
| Test: |  | | | |  |

**INTERVIEW QUESTIONNAIRE**

**ESTABLISHMENTS PRODUCING, PROCESSING AND TRADING STREET FOOD**

|  |  |
| --- | --- |
|  |  |

**PART I. GENERAL INFORMATION**

Please provide some information:

| **1.**Establishments of the type:  [ ] 1. Street food processing and trading establishments  [ ] 2. Establishments that only do street food business  [ ] 3. The facility only produces street food processing | |
| --- | --- |
| **2.**First and last name of the person who replied:  ............................................................................................... | **3.**Gender:  [ ] 1. Male [ ] 2. Female |
| **4.**Age:............................................  **5.**Permanent address:  [ ] 1. Ward: …………………………………  [ ] 2. district: ……………………………… | **6.**Ethnicity:  [ ] 1. Kinh  [ ] 2. Chinese  [ ] 3. Khmer  [ ] 9. Other: (Specify) ............................... |
| **7.**Religion:  [] 1. Buddhism  [] 2. Hoa Hao Buddhism  [] 3. Catholicism  [ ] 4. Protestant  [ ] 5. Cao Dai  [ ] 6. Non-religious  [ ] 9. Other (Specify):.......................................................... | **8.**Education level  [ ] 1. Uniterate  [ ] 2. Can read, write  [ ] 3. Level 1  [ ] 4. Level 2  [ ] 5. Level 3  [ ] 6. Intermediate/College  [ ] 7. University  [ ] 8. Post-university |
| **9.**Facility name:……………………………  **10.** Number of years of business:........................................  **11.**Facility address:  [ ] 1. Ward:..........................................................  [ ] 2. County:..............................................................  [ ] 3. Facility phone number:.................................  [ ] 4. Name of the facility owner:............................ | **12.**How many years have you worked in this field?  [ ] 1. Less than 1 year  [ ] 2. From 1 to less than 3 years  [ ] 3. From 3 to less than 5 years  [ ] 4. 5 years or more |

**PART II. KNOWLEDGE AND ATTITUDES TOWARDS STREET FOOD SAFETY**

| **1. HEALTH CHECK-UP**  **1..** Do you need a health check-up when you're in the food business? [ ] 1. Yes [ ] 2. No  **2.**Do you know how long it takes for a medical examination?  [ ] 1. 6 months  [ ] 2. 1 year  [ ] 3. 3 years  [ ] 98. Unknown  [ ] 99. Other (clearly stated):...........................................  **2. TRAINING**  **3..** Do you need to be trained in knowledge of food safety and hygiene?  [ ] 1. Yes [ ] 2. No  **4.** Do you know how many years of knowledge about traffic safety is valid?  [ ] 1. 1 year  [ ] 2. 3 years  [ ] 3. Indefinitely  [ ] 98. Unknown  [ ] 99. Other (clearly stated):...........................................  **3. BUSINESS PROCEDURES**  **5.** Do you think that the issuance of the certificate or the COMMITMENT to ensure traffic safety is necessary for the facility?  [ ] 1. Yes [ ] 2. No  **6.** Do you know how many years food safety certificates or pledges are valid?  [ ] 1. 1 year  [ ] 2. 3 years  [ ] 3. Indefinitely  [ ] 98. Unknown  [ ] 99. Other (clearly stated):............................. | **4. FOOD ATVS**  **7.**. Do you think keeping street food safety is important to your facility?  [ ] 1. Yes [ ] 2. No  **8.**What is the risk of contaminated food? **(multiple choices)**  [ ] 1. Chemicals  [ ] 1. Germs  [ ] 1. Glass, metal, hair, hair fragments  [ ] 1. Water  [ ] 1. Air  [ ] 1. From contaminated raw materials  [ ] 1. From the hands of contaminated business processing producers  [ ] 1. From insects, animals with pathogens  [ ] 1. From unsanitary storage and transportation equipment  [ ] 98. Unknown  [ ] 99. Other (Specify):.....................................................  **5. MATERIALS CONTAINING RAW MATERIALS AND FOOD DURING PROCESSING**  **9.**For raw and cooked food, how do you use processing tools to ensure cleanliness?  [ ] 1. Use a knife, separate cutting boards  [ ] 1. Use food utensils separately (chopsticks, spoons,...)  [ ] 1. Use the tool containing raw materials separately  [ ] 98. Unknown  [ ] 99. Other:............................................... |
| --- | --- |
| **6. WATER USE**  **10.** What is clean water according to you?  **(multiple choices)**  [ ] 1. Drinking water  [ ] 1. Treated well water ( settle, filter, disinfect)  [ ] 1. River and canal water, ponds and lakes  [ ] 1. Rainwater is properly treated and treated *(after a heavy rain about 15 minutes from the roof tiles, ton or treated concrete filtered with lime water or sand tank)*  [ ] 98. Unknown  [ ] 99. Other (Specify):......................................  **7. THE PERSON WHO PREPARES, DIRECT CONTACT WITH FOOD**  **14.**When did you wash your hands with soap?  [ ] 1. After going to the toilet  [ ] 2. Pre-processing  [ ] 3. Before dividing cooked food  [ ] 4. None of the above forms  [ ] 99. Other (Specify):..................................  **15.** If your hands are close, what should you do before processing or selling food?  **(multiple choices)**  [ ] 1. Bandaged  [ ] 1. Use gloves for processing, selling food  [ ] 1. Do not directly prepare, sell food  [ ] 98. Unknown  [ ] 99. Other (Specify):................................  **16.** When preparing food for guests Should you equip protective facilities? Like that?  **(multiple choices)**  [ ] 1. Wearing an apron  [ ] 1. Wearing a hat  [ ] 1. Wear a mask  [ ] 1. Wear gloves  [ ] 1. No equipment required  [ ] 98. Unknown  [ ] 99. Other:...................................................................  **17.** According to you, wearing an apron, wearing a hat works? **(multiple choices)**  [ ] 1. Beauty when serving  [ ] 1. Does not work  [ ] 1. Reduce the spread of pathogens from person to  food  [ ] 98. Unknown  [ ] 99. Other (Specify):............................  **8. HEIGHT OF THE PROCESSING PLACE**  **18.**What effect do you have onclean processing? **(multiple choices)**  [ ] 1. Reduce the risk of contamination of pathogens,  toxins into food  [ ] 1. Prevention of reproduction and development of  pathogens  [ ] 1. Avoid re-infection of food  [ ] 98. Unknown  [ ] 99. Other:...................................  **19.**What altitude should the food processing table be atcompared to the ground?  [ ] 1. On the table high > 60cm  [ ] 2. Close to the background  [ ] 98. Unknown  [ ] 99. Other (Specify):............................ | **11.** Do you need to use clean water according to your food washer?  [ ] 1. Yes [ ] 2. No  **12.** Do you need to wash dishes, bowls, forks, chopsticks, spoons?  [ ] 1. Every time I change it  [ ] 2. Several times instead of once  [ ] 3. Direct flushing  [ ] 99. Other (Specify):.................................  **13.** Does the pathogen ice exist?  [ ] 1. Yes [ ] 2. No  **9. RAW MATERIALS, ADDITIVES**  **20.** How often do you choose food ingredients for food processing? **(Suggested)**  **(multiple choices)**  [ ] 1. Raw materials are fresh, raw  [ ] 1. Have a clear origin  [ ] 1. Shelf life  [ ] 1. Cheap ingredients  [ ] 98. Unknown  [ ] 99. Other:.............................................  **21.** Should you use additives in food processing? (water coloring, welding, coloring,...)  [ ] 1. Yes [ ] 2. No  **If so,**how should it be used? **(multiple choices)**  [ ] 1. Included in the allow list  [ ] 1. Correct dosage  [ ] 1. Cheap type  [ ] 1. Eye-catching colored type  [ ] 1. What kind of crunchy food makes it  [ ] 98. Unknown  Other (Specify):.................................  **22.** Do you know prohibited acts in food production and business? **(multiple choices)**  [ ] Expired food business  [ ] Use of banned chemicals and additives for food processing  [ ] 98. Unknown  [ ] 99. Other:........................................  **23.** When do you usually go to buy food for processing?  [ ] 1. Morning  [ ] 2. Noon  [ ] 3. Afternoon  [ ] 4. Evening  **24.**. Where do you usually buy food and ingredients for processing? **(multiple choices)**  [ ] 1. Supermarkets  [ ] 1. Convenience store  [ ] 1. Market cage house  [ ] 1. Farm - Farmer's Household  [ ] 1. Small market (roadside market)  [ ] 1. Self-sed for self-sed  [ ] 99. Other:……………………… |
| **10. FOOD STORAGE CABINETS, SHIELDING**  **25.**Should you sell cooked food in a glass cabinet?  [ ] 1. Yes [ ] 2. No  **26.**What are the benefits of selling cooked food in glass cabinets? **(multiple choices)**  [ ] 1. Avoid contamination into food  [ ] 1. Avoid dust  [ ] 1. Against flies, stings, insect pests,...  [ ] 1. Does not work  [ ] 98. Unknown  [ ] 99. Other:......................................  **27.** Do you need to regulate food packaging?  [ ] 1. Yes [ ] 2. Not  **28.** What types can you use according to your food packaging? **(multiple choices)**  [ ] 1. Paper  [ ] 1. Plastic containers  [ ] 1. Banana leaves, lotus leaves  [ ] 1. Plastic bags  [ ] 99. Other:................................ | **11. TEMPERATURE AND STORAGE TIME**  **29.**At what temperature is it safe to store processedfood?  [ ] 1. <^5 0^C for cold food  [ ] 2. >^5 0^C for cold food  [ ] 3. > 60^0^C for hot food  [ ] 4. < 60^0^C for hot food  [ ] 99. Other (Specify):.............................  **30.** What measures are used to kill common pathogenic bacteria?  [ ] 1. Use high heat (cook at boiling temperature for at least 3 minutes)  [ ] 2. Use low temperatures (from 0 to 5 degrees Celsius)  [ ] 98. Unknown  [ ] 9. Other (Specify):..........................  **31.**According to you the maximum time for food to ripen at a temperature **is** usually what time :....................................  [ ] 98. Unknown |
| **12. FOOD POISONING**  **32.** Can you state the possible causes of food poisoning? **(multiple choices)**  [ ] 1. Germs  [ ] 1. Chemicals  [ ] 1. Food with available toxins  [ ] 1. Food is transformed  [ ] 98. Unknown  [ ] 99. Other:...................................  **33.** What are the main symptoms of food poisoning?  **(multiple choices)**  [ ] 1. Abdominal pain [ ] 1. Nausea  [ ] 1. Diarrhea [ ] 1. Fever  [ ] 1. Vomiting [ ] 1. Dizziness  [ ] 1. Angina [ ]1. Flushing face  [ ] 98. Unknown  [ ] 99. Other:..................................  **34.** What are the consequences of non-compliance with traffic safety regulations? **(multiple choices)**  [ ] 1.Risk of food poisoning  [ ] 1.The facility is suspended  [ ] 1.Sanctioned facilities  [ ] 98. Unknown  [ ] 99. Other:...................................  **35.** If your customer has food poisoning, what responsibility do you have?  ................................................................. | **13. GARBAGE AND WASTE DISPOSAL**  **36.**According to you, does the processing ground need to be cleaned after processing? [ ] 1. Yes [ ] 2. No  **37.**Do you need trash cans in the diner? [ ] 1. Yes [ ] 2. No  **If so,** how to collect garbage?  [ ] 1. One session  [ ] 2. One day  [ ] 3. A week  [ ] 4. Fill when filled  [ ] 98. Unknown  [ ] 99. Other: .......................................................................  **38.** According to you waste containers, garbage works? **(multiple choices)**  [ ] 1. Anti-pollution  [ ] 1. Ensuring beauty  [ ] 1. Fight flies, bugs  [ ] 1. Unnecessary  [ ] 98. Unknown  [ ] 99. Other:....................................  **39.** In what form should you be treated if the food is not safe?  [ ] 1. Re-processing  [ ] 2. Destruction  [ ] 3. Transfer of use purposes (as food for livestock, poultry, fish,...)  [ ] 4. Leave  [ ] 99. Other:....................... |

**PART III. STREET OSH PRACTICE**

| **1.** Number of years of business..............................................................  **2.**Total business capital (time of survey):…………………(million) | | | | | **3.** The facility belongs to:  [ ] 1. Fixed  [ ] 2. Mobile  **4.**Total area to use..................... (m2) | | | |  |
| --- | --- | --- | --- | --- | --- | --- | --- | --- | --- |
| **1. BUSINESS PROCEDURES**  **5.**Business license: [ ] 1. Yes [ ] 2. No  **6.**Do you currently have the following certificates? | | | | | | |  | | |
| [ ] 1.Certificate of eligibility  [ ] 1.Certificate of food safety knowledge  [ ] 1.Medical certificate | [ ] 1. Yes [ ] 2. No  [ ] 1. Yes [ ] 2. No  [ ] 1. Yes [ ] 2. No | | | | | [ ] 1. Still valid [ ] 2.out of date  [ ] 1. Still valid [ ] 2.out of date  [ ] 1. Still valid [ ] 2.out of date | | |  |
| **7.**Production/business items of the establishments (observe)  **(multiple choices)**  [ ] Food has water (vermicelli, noodles, noodles, noodles, porridge ...)  [ ] Rice, sticky rice  [ ] Food without water (wet cake, rice cake,...)  [ ] Takeaway food (bread, dumplings,...)  [ ] Cold food (spring rolls, rolls, cold meats,...)  [ ] Ice cream, juice and the like  [ ] Café, soft drinks  [ ] Tree stones, ice stones  [ ] Pub (beer)  [ ] Groceries (department, cropped goods,...)  [ ] Other:.............................................................................  **8.**Processing type (processing production)/feed business of the facility: **(multiple options)**  [ ] 1. On-site processing and trading  [ ] 1. Selling ready-to-eat food  [] 1. Processed from elsewhere  [ ] 1. Processing to bring to other places for sale  [ ] 99. Other (Specify):........................................................  **9.** Time of food production/trading of establishments:  [ ] 1. All day (morning to evening)  [ ] 2. Early morning  [ ] 3. Noon  [ ] 4. Evening  [ ] 9. Other (Specify):........................................................... | | **2. THE PERSON WHO PREPARES, DIRECT CONTACT WITH FOOD**  **10.** People processing/selling existing food:  **(multiple choices)**  [ ] 1. Wearing an apron  [ ] 1. Wearing a hat  [ ] 1. Wear a mask  [ ] 1. Wear gloves  [ ] 1. Smoking  [ ] 1. Neat, clean clothes  [ ] Other:.............................................  **11.** How is the hand washing of food institutions/sellers? **(multiple choices)**  **(n= number of observations)**  [ ] 1. Wash your hands when starting work  [ ] 1. Wash your hands after processing  [ ] 1. Wash your hands after going to the toilet  [ ] 1. Wash your hands before contact with food  [ ] 1. Wash your hands with soap  [ ] 1. Dry your hands after washing your hands  [ ] 1. Other (clearly stated):.....................................  **12.** The condition of the hand of the processing /seller food?  [ ] Short nails  [ ] Long nails  [ ] Wearable hand jewelry (rings, watches, rings,...)  [ ] Other (Specify):....................................................  **13.** Does the person processing/selling food have skin diseases?  [ ] 1. Yes [ ] 2. No | | | | | | |  |
| **3. INFRASTRUCTURE (observe)**  **14.** The facility has:  [ ] Home  [ ] Toilets  [ ] Food outlets are located > 60 cm above the ground  [ ]The facility has a one-way kitchen  [ ]Where to wash your hands [ ] Soap [ ] Hand wipes | | [ ]Where to wash tools  [ ]The production and business area has a separate source of pollution such as toilets, sewers  **15.** Is it clean? [ ] 1. Yes [ ] 2. No  **16.** What is the ta processing place of the facility?  [ ] 1. On the table > 60cm  [ ] 2. On the floor  [ ] 99. Other : .......................................................... | | | | | | |  |
| **4. WATER USE (observe)**  **16.** Is there water use?  [ ] 1. Yes [ ] 2. No  **17.** Water used:  [ ] 1. Drinking water  [ ] 2. Treated well water  [ ] 3. River and canal water, ponds and lakes  [ ] 4. Rainwater is properly treated and treated  [ ] 6. Other :..........................................................................  **18.** Does the facility have enough water to use?  [ ] 1. Yes [ ] 2. No  **19.** Does the facility have a refrigerator, refrigerated container (ice bucket)? [ ] 1. Yes [ ] 2. No | | **6. MATERIALS CONTAINING RAW MATERIALS AND FOOD BEFORE AND AFTER PROCESSING (observe)**  **22.** Facilities have:  [ ]Separate tools and equipment to accommodate food before and after processing  [ ]Eating utensils, food packaging of origin, licensed  [ ]Food and drinks after processing are left in glass cabinets or have hygiene shielding  [ ]Live and primary food for  **23.** What are the facilities currently using the finished product package? **(multiple choices)**  [ ] 1. Newspapers  [ ] 1. Removal paper  [ ] 1. Plastic containers  [ ] 1. Banana leaves, lotus leaves  [ ] 1. Plastic bags  [ ] 1. Other (Specify):................................................  **24.** Does the existing facility perform the contents after processing? **(multiple choices)**  [ ] 1. Use a separate cutting knife for cooked raw food  [ ] 1. Use chopsticks, sandwiches to scoop up cooked food  [ ] 1. Use gloves to divide food  **7. GARBAGE AND WASTE TREATMENT**  **25.** Does the existing facility treat waste?  **(multiple choices)**  [ ] 1. Garbage (trash)  [ ] 1. Waste estimates  [ ] 1. Daily processing  **26.** For frying oil, how does the facility handle it?  Do you use it or not?  Ask  [ ] 1. Where to buy:.................................................  [ ] 1. Have sheep fry again and again  [ ] 1. Treatment of used oil:...................................  **27.** Do you save samples at the facility?  [ ] 1. Yes [ ] 2. No | | | | | | |  |
| **5. RAW MATERIALS**  **20.** Does the purchased base food material originate? **(multiple choices)**  [ ] 1. Contracts  [ ] 1. Invoices for documents  [ ] 1. Contracts, invoices  [ ] 1. Only books, prescription notes  [ ] 1. Unknown origin  [ ] 1. Food ingredients are not overdue, not deformed  [ ] 1. Meat and processed products from un quarantined meat  [ ] 1. Records of the origin of raw materials  [ ] 99. Other (Specify):.........................................................  **21.** In case of unknown origin, specify the type of raw material:  ……………………………………………………………………………………………………………………………… | |  |  |  |  |  |  |  |  |
|  | | |  |  | | |  |  | |

**PART IV. Human resources and training (For establishments with a number of employees of 2 or more)**

*Write equivalent number in cell*

| Total number of employees | Gender | | Age group | | | Have been trained in food safety and hygiene | |
| --- | --- | --- | --- | --- | --- | --- | --- |
|  | Male | Female | <15 | 15-55 | >55 | Have | Not |
|  |  |  |  |  |  |  |  |

| Education level  Uniterate  Level 1  Level 2  Level 3  Intermediate/College  University  Post-university  Other (specify):.................................................................. | ......  .....  ......  ......  ....  .....  .....  ...... |
| --- | --- |

**Do you propose to improve the situation of food safety and hygiene at the facility?**

......................................................................................................................................................

......................................................................................................................................................

......................................................................................................................................................

......................................................................................................................................................

**Thank you for your interview !**

| **Interviewer:**..........................................................................................................  **Interview date:** .................................................................................................. |
| --- |

***Table S3: The content of the 10 food hygiene and safety criteria***

| **Criteria** | **Content** |
| --- | --- |
| 1. Have adequate safe water | Make sure there is sufficient clean water and ice, refrigerators, cold storage containers. |
| 2. Separate raw and cooked food (use separate utensils for food handling, store separately) | Separate tools, containers and display areas for raw and cooked foods; use separate knives and cutting boards for raw and cooked foods; use chopsticks and tongs to pick up cooked food; use gloves to handle food. |
| 3. Keep food vending/preparing site clean, away from rubbish, toilets, open drains and animals | Food processing places must be clean, separated from sources of pollution (sewers, garbage, sanitation facilities, livestock and poultry sale points); one-way kitchen model is applied. |
| 4. Have periodic medical examination (and get certified) | Food processing personnel must receive periodic physical exams at least once a year and obtain the physical exam certificate. |
| 5. Get training on food safety and hygiene (and get certified) | Food processing personnel must have a certificate of food safety and hygiene training. |
| 6. Wear protective clothes (apron, mask, hat) when preparing and selling food. Wash hand before and after processing food and after going to the toilet | Food processing/selling personnel must dress neatly with clean protective clothing such as apron, hat, mask; they have to remove all jewelry, keep fingernails and hands clean at all times; they have to wash hands before and after processing food and after going to the toilet. Use soap for washing hands, dry hands after washing; they must not have skin diseases. |
| 7. Provide proof of origin for ingredients, food additives and pre-prepared food. Only use food that are authorized and use with allowable amount | Food ingredients must be from known and safe origin; food vendors must be able to provide proof of origin (contract, receipt). Food ingredients must not exceed the expiry date, not be denatured; Meat and meat products are separated; record origins of ingredients. |
| 8. Prepare and/or store food on tables or shelf at least 60cm high from the ground to protect them from dust, insects, dirt and direct sun | Food displayed for sale must be placed on a table or shelf at least 60 cm above the ground. |
| 9. Place food for sale in glass cabinets to protect them from dust, insects, dirt and direct sun | Food displayed for sale must be kept in glass cabinets or hygienic storage containers. |
| 10. Have enough waste bins with lids and empty waste on a regular basis | Waste containers must have lids; empty waste on a regular basis; do not reuse oil. |
